# New strategies of clinical precision medicine

**DOI:** 10.1002/ctm2.135

**Published:** 2022-03-01

**Authors:** Xiangdong Wang

**Affiliations:** ^1^ Department of Pulmonary and Critical Care Medicine, Zhongshan Hospital Fudan University Shanghai Medical College Shanghai China

The rapid development of technology has facilitated the growth of precision medicine as an efficient therapy that is increasingly applied in clinical practice, including monogenetic disorders, cancer, metabolic dysfunction, infections, and chronic disease. The words “precision medicine” and “clinical precision medicine” can be searched in PubMed databases from 1952 and 1975, respectively. The number of published articles on “precision medicine” and “clinical precision medicine” shows an upwards trend from 2010 and 2015, respectively, of which most consisted of reviews, commentaries, and communications. In 2012, Dolsten and Søgaard defined the concept of precision medicine as an approach to improve the outcomes for patients through the discovery and development of target‐based medicines and vaccines, and to facilitate clinical practice of personalized medicine through the integration of clinical and molecular information.^1^ Precision medicine is an emerging discipline to develop new therapies for a clear target group of patients on basis of clinical and molecular phenomes and characters, subpopulations and susceptibilities, and heterogeneities and responses to treatment of the individual.

It is crucial to highlight the importance of strategies and strategic applications in the practice of precision medicine, in addition to biomarker discovery and development, technologies, databases, regulations, commercial values, and bioethics. Precision medicine is tailored to each individual treatment plan based upon clinical decision‐making, in contrast to evidence‐based medicine generalized for all patients.^2^ However, the application of precision medicine remains limited due to the inherent limitations of randomized control trials. Significant research has been dedicated to developing new strategies for applying clinical precision medicine and how to accelerate and smoothen the transition from research‐based investigations and scientific theories into clinical trials and practices, while remaining financially viable for market production with increasing magnitude. The aim of this editorial is to call special attentions on the recent development of new strategies for clinical precision medicine and discuss potential values of new methodologies for measurements and analyses for clinical practice, for example, pharmacogenomics, clinical phenomics, clinical bioinformatics, DNA/RNA sequencing, single cell biomedicine, gene editing, and clinical trans‐omics.

Pharmacogenomics is an approach to clinical precision medicine by defining and selecting target‐based drug categories, doses, administrations, and combinations to maximally reduce toxicity and remain efficacy. Genetic phenotypes can be altered in response to drugs and play the decisive role in the severity and phenome of drug response. Clinical pharmacogenomics experienced phases of coding and noncoding region variants in response to drugs, large‐scale gene study/genome wide association study, and tumor RNA/DNA sequencing. With the rapid development of gene sequencing, a more comprehensive understanding of germline and somatic gene changes, mutation, and heterogeneity in cancer has provided new insights for defining the role of pharmacogenomics in precision medicine.^3^ Although challenges exist, gene‐based clinical pharmacogenomics testing has the potential to be a novel approach that can direct the therapeutic and monitoring strategy of precision medicine for patients. A precise self‐validation system was proposed as a new approach of precision medicine to screen and optimize therapeutic strategies of individual's treatment by scanning the targeted drug efficacy and specificity in the patient's own cancer cells.^3^ A protein structure–guided compound screening or a DNA‐encoded chemical library based on pharmacogenomic profiles, gene mutations, self‐validation systems, and heterogeneity could prove beneficial in the development of clinical precision medicine.

Mutation‐based therapy is an important strategy in clinical precision medicine that provides a gene sequencing indicated therapeutic direction, germline/somatic mutation‐guided clinical protocol, pharmacogenomics‐supported drug selection, and expert‐decided therapy. Astras et al treated patients with progressive cancer and failing at least one standard of care treatment using the precision medicine strategy on basis of putative somatic mutations, immunohistochemistry, and molecular profiles.^4^ The strategy is dependent on target‐specific drug selections and pharmacogenomics‐driven recommendations regarding mutations, heterogeneity, and molecular profiles. The efficacy of precision medicine strategy should be monitored by disease‐specific biomarkers with specificities of disease duration, stage, severity, and drug response.^5^, ^6^ Laes et al analyzed the molecular profiles of 1057 advanced cancer patient samples after failing at least one standard of care treatment and discovered that the combination of next‐generation sequencing, immunohistochemistry, and other specific tests provided better information compared to utilizing each method separately.^7^ The performance of precision medicine strategy is highly correlated to the effort and understanding the clinician possesses with regard to the strategy, the availability and cost of target drugs, and the cancer types and severities. Of those influencing factors, clinicians play the most important role as the central commander in the performance of clinical precision medicine, for example, patient selection, disease definition, organization of multidisciplinary discussions, interpretation of gene sequencing data, and decision‐making of gene sequencing panels, pharmacogenomic identification, and precision medicine strategy. The selection of strategy is a critical step as it is frequently the limiting link in precision medicine and has a direct impact on the outcome for the patient. It is suggested that the participation of clinical pharmacologists is required to discuss mutation landscapes during data analyses and to contribute to the selection of therapeutic strategies during decision‐making.

An important criterion for selecting the most suitable precision medicine strategy is how much the severity of the disease, clinical phenomes, and the survival time of patient can be improved. The strategy should be as simple, definitive, and efficient as possible. The approach of “Zhongshan Strategy” in clinical precision medicine consists of mutation‐based therapy for patients with failure of chemotherapy, radiotherapy, and first‐line therapy, computational simulation of drug screening, drug reference of DNA library, patients own cell screening of target‐based therapies, and patient disease models.^3^ This approach requires a comprehensive understanding of the heterogeneity mechanisms and provides a more precise system for selection and validation. The applicability, efficiency, and feasibility of such an approach need to be weighted together with a higher requirement for technology and increased cost. Qian et al recently presented a multidisciplinary therapy strategy for precision medicine (MDTS‐PM) to gather multidisciplinary experts and develop a real‐time therapeutic strategy by integrating clinical phenomes, patient response to drugs, and gene sequencing of tissue DNA and circulating DNA.^8^ Target‐specific therapies in the combination of clinical phenomes, genetic information, diagnosis, and treatment were dynamically adjusted on basis of the progression of the patient with advanced metastasis in multiple organs/tissues after failed treatment with various single or combined therapies. This approach requires to organize a stable team with knowledgeable experts (e.g., oncologist, pathologist, geneticist, radiologist, pharmacologist, bioinformatician, and nurse), collect clinical and molecular phenomes with the digital evaluation score system, have an automatic recording, updating, and communication system among both experts and between clinician and patient, as well as design a multitargets‐based therapy, as detailed previously.^9^, ^10^, ^11^, ^12^, ^13^ MDTS‐PM is more suitable for patients with advanced cancer, complex drug resistance, rapid progression, comprehensive gene profiles, and tumor complicated with multiple organ metastasis at the later phase. Dynamic monitoring of clinical phenotypes and gene mutation profiles in patients is a critical part in the development of new strategies for clinical precision medicine.

The precision medicine is a part of genomic medicine and clinical and translational medicine to translate mutation‐based or sequencing‐evidenced therapy and strategy into clinical practice, monitor the disease progression using gene‐based biomarkers, predict the patient prognosis using transcriptional networks and interactions, prevent the disease occurrence using germline heterogeneity and genetic background, integrate gene mutations with pharmacogenomic profiles, and optimize the strategy of precision medicine for individuals. In addition to therapeutic strategies, precision medicine will be a new approach for health care and disease prevention in larger populations. The process of translating clinical precision medicine into new regulations and policies has been initiated, for example, molecular testing guideline for target‐based therapies in patients with cancer.^14^ The intercommunication and interaction between immune and metabolic checkpoints and gut microbiota were suggested as a source to generate new therapeutic targets and could aid in developing new strategies.^11^, ^15^ Clinical trans‐omics is a new approach that can integrate molecular multi‐omics with clinical phenomics and can present the full landscape of patient phenome‐based molecular networks to identify diagnostic biomarkers and therapeutic targets.^13^ Clinical trans‐omics as a systemic and comprehensive discipline can be critical in identify new strategies for precision medicine on basis of targets crossing multidimensional networks from molecular multi‐omics. Thus, bioinformaticians and pharmacogenetic experts play crucial roles in the MDTS‐PM team and in the decision‐making of precision medicine. New strategies of precision medicine will be the important member of clinical and translational medicine, and need to be further developed and validated in clinical basket and/or umbrella trails for clinical practice.
